# TFAP2A promotes NSCLC malignant progression by enhancing AOC1 transcription

**DOI:** 10.1186/s41065-025-00524-2

**Published:** 2025-08-14

**Authors:** Xiang Miao, Hongzhen Zheng, Huimin Mo, Jing Chang, Qin Jia, Hai Zhou

**Affiliations:** Department of Pulmonary and Critical Care Medicine, Shidong Hospital of Yangpu District, No. 999, Shiguang Road, Yangpu District, Shanghai, 200438 China

**Keywords:** Non-small cell lung cancer, AOC1, TFAP2A, Malignant progression, Transcriptional regulation

## Abstract

**Background:**

Non-small cell lung cancer (NSCLC) has high mortality, and patients show variable outcomes and drug responses. Amine oxidase copper-containing 1 (AOC1) is considered an oncogene in many types of tumors. Transcription factor AP-2 alpha (TFAP2A) can affect a variety of biological processes and play a crucial role in driving tumorigenesis and tumor development. Consequently, this work is designed to delve into the effects of AOC1 and TFAP2A on NSCLC progression.

**Methods:**

Bioinformatics analysis was employed to analyze AOC1 and TFAP2A expression in the TNMplot database and the survival significance of high- or low-expression of AOC1 in NSCLC patients. Quantitative real-time polymerase chain reaction (RT-qPCR) and western blot were implemented to assay the mRNA and protein expression levels of genes. Cell proliferation, migration, and apoptosis were detected using 5-ethynyl-2’-deoxyuridine (EdU), wound healing, and flow cytometry, respectively. In addition, mitochondrial membrane potential and reactive oxygen species (ROS) were examined using JC-1 and ROS detection kits. The macrophage M2 polarization was tested via flow cytometry. The construction of subcutaneous transplanted tumors in nude mice confirmed the effect of AOC1 in vivo. In order to discern the upstream regulatory mechanisms, the JASPAR database was utilized to predict the transcription factors and binding sites associated with AOC1. Chromatin immunoprecipitation (CHIP) and luciferase reporter gene assays were performed to solidify the binding relationship between AOC1 and TFAP2A.

**Results:**

AOC1 and TFAP2A levels were increased in NSCLC tumor tissues and cell lines (A-549 and NCI-H1299). AOC1 knockdown inhibited NSCLC cell proliferation, migration, M2 macrophage polarization, and mitochondrial membrane potential, and facilitated cell apoptosis and ROS. In vivo, sh-AOC1 suppressed NSCLC tumor growth. Furthermore, TFAP2A facilitated AOC1 expression via transcriptional regulation in NSCLC. Mechanically, TFAP2A promoted NSCLC progression via facilitating AOC1 expression.

**Conclusions:**

Silencing TFAP2A inhibits NSCLC progression via regulating AOC1 transcription. Hence, AOC1/TFAP2A may be a feasible therapeutic target for NSCLC.

**Graphical Abstract:**

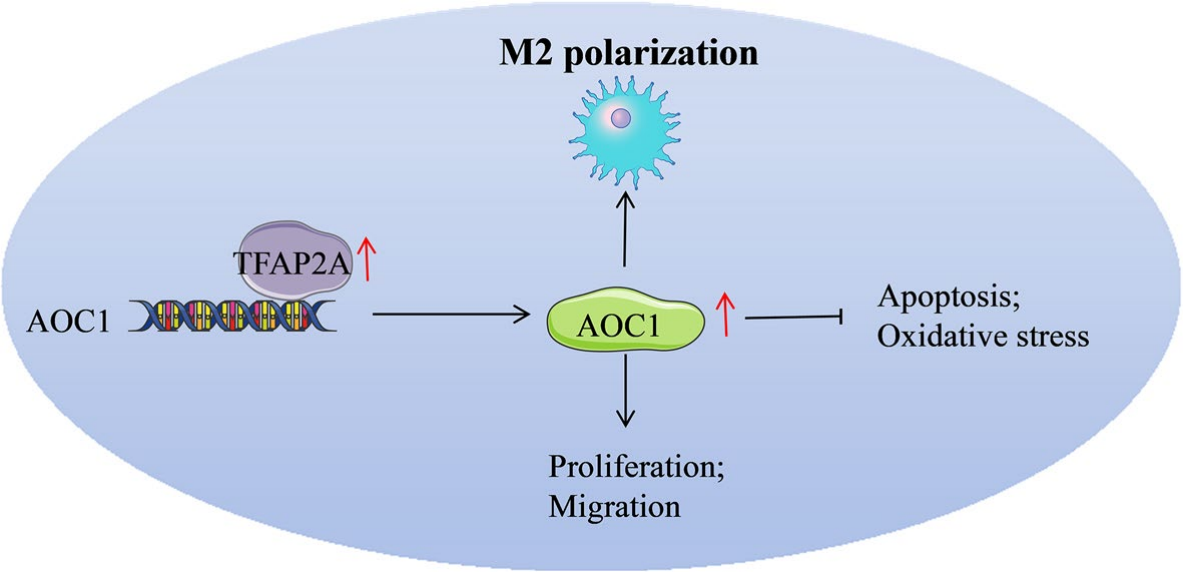

**Supplementary Information:**

The online version contains supplementary material available at 10.1186/s41065-025-00524-2.

## Introduction

Non-small cell lung cancer (NSCLC) (a heterogeneous disease), accounting for approximately 85% of all lung cancer cases, remains a leading cause of cancer-related mortality globally [1, 2]. In NSCLC diagnosis, the majority of patients are diagnosed at a late stage, resulting in a low five-year survival rate [3]. Notably, mediastinal lymph node metastasis predicts prognosis in non-metastatic NSCLC [4]. Surgical intervention, coupled with adjuvant chemotherapy and radiation before or after surgery, has been designed to improve cure rates and prolong survival in patients with NSCLC [5]. However, recurrence, metastasis, and adverse side effects are the limitations of this approach, and it is important to explore new potential molecular targets to promote the treatment of NSCLC.

Amine oxidase copper-containing 1 (AOC1), a secreted glycoprotein, can catalyze the putrescine and histamine degradation [6]. Additionally, AOC1 is involved in regulating many biological processes, such as tumor growth and development, and neoplasia [7]. For instance, Xu et al*.* demonstrated that downregulation of AOC1 repressed human gastric cancer (GC) cell invasion, migration, and proliferation, and elevated cell apoptosis [8]. AOC1 promoted malignant features of hepatocellular carcinoma (HCC) cells, including proliferation, migration, and invasion [9]. However, the potential epigenetic regulation of AOC1 in NSCLC pathogenesis has not been reported.

Transcription factor AP-2 alpha (TFAP2A) is a member of the transcription factor AP-2 family and has been implicated in various pathophysiological processes, including tumors [10]. TFAP2A knockdown hindered cervical cancer cell proliferation and migration, and facilitated apoptosis [11]. A study had shown that in oral squamous cell carcinoma (OSCC), TFAP2A was elevated, and NFκB (RelA) enhanced OSCC progression via regulating TFAP2A-Wnt/β-catenin signaling [12]. Besides, TFAP2A accelerated the progression of lung adenocarcinoma (LUAD) via promoting immune-associated gene integrin β4 [13]. Guoren et al*.* reported that TFAP2A could promote LUAD metastasis via positively mediating Inositol-trisphosphate 3-kinase A transcription [14]. Besides, TFAP2A could activate high mobility group AT-hook 1 to contribute to LUAD progression [15]. To sum up, TFAP2A is associated with NSCLC progression.

Thus, the aim of this study is to investigate the action of AOC1 and TFAP2A on the malignant behaviors of NSCLC. Our findings in this study will provide a reference for future research and clinical application of NSCLC pathogenesis.

## Materials and methods

### Data sources

The clinical data for NSCLC was obtained from the TNMplot database. TNMplot was used to analyze gene expression across 56,938 unique samples, including 15,648 normal tissues, 40,442 primary tumors, and 848 metastases sourced from Gene Expression Omnibus (GEO), Genotype Tissue Expression Project (GTEx), Cancer Genome Atlas (TCGA), and TARGET databases.

### Clinical specimens

The NSCLC tissues (*n* = 89) and adjacent non-cancer tissues (*n* = 89) were obtained when patients with NSCLC were treated with traditional surgery and were taken with the informed consent of both patients. This research was reviewed and authorized by Shidong Hospital of Yangpu District.

### Quantitative real-time polymerase chain reaction (RT-qPCR)

In this research, the collected NSCLC tissues and cells with different treatments were added with Trizol reagent (Jude Antai Technology Co., Ltd., Beijing, China) to extract total RNA. Subsequently, reverse transcription in this experiment was carried out using PrimeScript™ RT reagent Kit (Takara, Osaka, Japan). The SYBR Green PCR Master Mix (Biosharp, Beijing, China) was used for the RT-qPCR reaction. Besides, forward and reverse primers employed for RT-qPCR were as follows (5’−3’): TFAP2A, CACCTAGCCAGGGACTTTGG and CAGCAGGTCGGTGAACTCTT; AOC1, GCTGCGGACAACTTCAACTG and CGGTAGTGCACCAAGTGAGT; β-actin, AGGATTCCTATGTGGGCGAC and ATAGCACAGCCTGGATAGCAA.

### Western blot

The total proteins in this research were extracted from NSCLC tissues and treated cells using RIPA lysis buffer. The proteins were separated using polyacrylamide gel electrophoresis. Next, the total proteins were transferred onto polyvinylidene fluoride (PVDF) membranes. After sealing fluid (Biosharp) was performed to enclose the membranes for 1 h, the membranes were combined with primary antibodies (AOC1 (ab278497, 1:1000, Abcam, Cambridge, MA, USA), TFAP2A (PA5-17,359, 1:1000, Invitrogen, Carlsbad, CA, USA), and β-actin (ab8226, 1:3000, Abcam)) at 4℃ for 12 h. Following that, the membranes were incubated with secondary antibodies at 37℃ for 2 h. Finally, an enhanced chemiluminescence system (JIAPENG, Shanghai, China) was used to analyze the protein levels. In this study, secondary antibodies were used as follows (obtained from Abcam): anti-rabbit (1:5000, ab6721) and anti-mouse (1:5000, ab6728).

### Cell culture and treatments

16HBE cells were provided by Yaji Biotechnology Co., Ltd. (Shanghai, China). Besides, human NSCLC cell lines (A-549 and NCI-H1299) were acquired from Procell Life Science & Technology Co., Ltd. (Wuhan, China). The RPMI-1640 medium (Yuchun Biotechnology Co., Ltd., Shanghai, China) containing 10% fetal bovine serum (FBS; Beyotime, Shanghai, China) was employed to culture these cells. These cells were incubated in CO_2_ incubators at 37℃.

### Knockdown and overexpression vector construction and transfection

The lentiviral vectors of short hairpin (sh) RNA negative control (sh-NC) and shRNA targeting AOC1 (sh-AOC1) and TFAP2A (sh-TFAP2A) were designed by RiboBio (RiboBio Inc., Guangzhou, China). AOC1 and TFAP2A overexpression vectors were also generated from Genewiz (Nanjing, China). Lipofectamine 2000 (Invitrogen) was used to transiently transfect according to the instructions of the manufacturer.

### Detection of proliferation

Cell proliferation in this research was measured using 5-ethynyl-2’-deoxyuridine (EdU) assay kit (RiboBio Inc.) following the guidelines of the manufacturer. In short, cells were plated in 12-well microplates and then transfected with shRNAs or plasmids. Cells were collected and fixed with paraformaldehyde (4%). After 0.5 h, Triton X-100 was added into each well, and the cells were reacted with Apollo staining reaction solution for 0.5 h. Lastly, the DAPI was used to stain the cell nucleus, and then the ability of cell proliferation was estimated using a fluorescence microscope.

### Wound healing assay

For the migration assay, A-549 and NCI-H1299 cells were seeded into 6-well microplates and transiently transfected with shRNAs or plasmids. The next day, the sterile pipette was employed to make the scratch. Finally, the results of migration were analyzed using an inverted microscope at 0 and 24 h.

### Detection of apoptosis

In this study, the Annexin V-FITC Apoptosis Detection Kit (Vazyme, Nanjing, China) was performed to estimate cell apoptosis. In short, the A-549 and NCI-H1299 cells were cultured in 12-well microplates. Subsequently, the cells were transiently transfected with shRNAs or plasmids, and then the cells were obtained and stained using Annexin V-FITC and PI (5 µL) at 37℃ for 0.25 h in a dark environment. The flow cytometry assay system was used to detect cell apoptosis.

### Detection of mitochondrial membrane potential and reactive oxygen species (ROS)

In this study, the mitochondrial membrane potential and ROS were detected using JC-1 and ROS assay kits (Beyotime) according to the protocol of manufacturer.

For the mitochondrial membrane potential assay, the cells with shRNAs or plasmid transfection were collected and re-suspended. Following that, the JC-1 staining working solutions were mixed with the cell suspension and reacted in an incubator with 5% CO_2_ for 20 min at 37℃. After centrifugation, the cellular supernatant was discarded, and then the cells were washed with JC-1 dye buffer (1 ×) two times. Finally, JC-1 dye buffer (1 ×) was used to resuscitate the cells, and then flow cytometry assay system was employed to examine the mitochondrial membrane potential.

After transfection with shRNAs or plasmids, the cells were collected. Subsequently, the cells were re-suspended with diluted DCFH-DA and incubated at 37℃ in an incubator with 5% CO_2_ for 20 min. These cells were washed using medium without FBS three times. Finally, the ROS levels were measured using a flow cytometry assay system.

### Detection of M2 polarization marker CD206^+^ cell rate

THP-1 cells (Bolson Biotechnology Co., Ltd., Shanghai, China) in this study were incubated in RPMI-1640 complete medium (Yuchun Biotechnology Co., Ltd.) with β-mercaptoethanol (0.05 mM) (Yubo Biotechnology Co., Ltd., Shanghai, China), which were stimulated with phorbol 12-myristate 13-acetate (PMA) (50 nm; Selleck, Shanghai, China) for 24 h to induce M0 macrophages. After A-549 and NCI-H1299 cells were transfected with shRNAs and/or plasmids, the conditioned medium (CM) was collected and was used to culture PMA-induced THP-1 cells. Following that, the cells were collected and combined with anti-CD163 (ab182422, 1:60, Abcam), and then the M2 polarization marker CD206^+^ cell rate was analyzed following the instructions of the manufacturer under flow cytometry assay system.

### Xenograft study

In this animal experiment, the BALB/c nude mice (6 weeks old) were provided by Weitonglihua (Beijing, China). The A-549 cells were transfected with lentivirus vectors containing sh-NC, sh-AOC1, vector, or AOC1, and then the cells were collected and re-suspended. The cells with different treatments were subcutaneously injected into BALB/c nude mice and named sh-NC, sh-AOC1, vector, and AOC1 groups. After 1 week, the tumor volume was detected, and then the tumor size in all nude mice was detected every 7 days. After 4 weeks following inoculation, the nude mice were killed, and the tumors were collected, photographed, and weighed. The partial tumors were used to perform an immunohistochemistry assay (IHC). This animal experiment was allowed by the Animal Care and Use Committee of Shidong Hospital of Yangpu District.

For the IHC assay, the tumor tissues were fixed, paraffin-embedded, and cut into tissue Sects. (4 µm). The sections were dewaxed and rehydrated. Following that, Sects. (4 µm) were processed with H_2_O_2_ (3%) for 10 min and placed in citrate buffer at microwave temperature for antigen repair. The goat serum was employed to block, and then the sections were combined with anti-AOC1 (ab278497, 1:2000) and anti-Ki-67 (ab279653, 1:1000) (obtained from Abcam) for 12 h at 4℃. The Sects. (4 µm) were combined with anti-rabbit (1:5000, ab6721) or anti-mouse (1:5000, ab6728) (obtained from Abcam) for 1 h at 37℃. The DBA was used to stain the sections after being washed. Next, the Sects. (4 µm) were counterstained with hematoxylin, differentiated with ethanol hydrochloride, dehydrated, and cleared. Finally, the sections were sealed with neutral gum, and the levels of AOC1 and Ki-67 were analyzed using a microscope.

### Chromatin immunoprecipitation (CHIP)

The EZ-ChIP Kit (Millipore, Billerica, MA, USA) was used for CHIP assay, and the detailed procedures were performed following the guidelines of manufacturer. In short, the cells were plated in a 10-cm plate. The formaldehyde (1%) was employed to fix the cells, and then the glycine was added to neutralize unreacted formaldehyde. Following that, the cells were lysed and collected. Cross-linked protein-DNA was immunoprecipitated with magnetic beads and combined with anti-TFAP2A (ab108311, 1:20, Abcam) rotated overnight at 4℃ for non-specific binding, and the normal IgG was used as a control. Small beads containing a protein-DNA complex were pulled down. The purified protein-DNA complexes in this experiment were reacted with proteinase K. RT-qPCR was used to analyze AOC1 expression.

### Dual luciferase reporter gene assay

The JASPAR database (http://jaspar.genereg.net/) in this research was used to analyze the TFAP2A-binding site in the AOC1 promoter. Mutant-type (mut) and wild-type (wt) reporter plasmids of AOC1 were amplified and cloned into pmirGLO vectors (Zeye Biotechnology Co., Ltd., Shanghai, China). The site-directed mutagenesis kit (Stratagene, CA, USA) was carried out to mutate TFAP2A binding site in AOC1 promoter. Following that, the cells were transiently transfected with sh-TFAP2A (TFAP2A) or sh-NC (vector) and AOC1-wt or AOC1-mut. Finally, the dual luciferase reporter gene assay system was employed to examine luciferase activity.

### Data analysis

The data in this research were presented as the mean values with standard error of mean, and *P* < 0.05 was regarded as significant. The GraphPad Prism 8 software (GraphPad Software, Boston, MA, USA) was used to make graphs and analyze data. The differences among multiple groups were analyzed using analysis of variance (ANOVA) followed by Tukey’s test, and Student’s *t* test was performed for analyzing the differences between two groups.

## Results

### AOC1 levels are increased in NSCLC

**Fig. 1:**
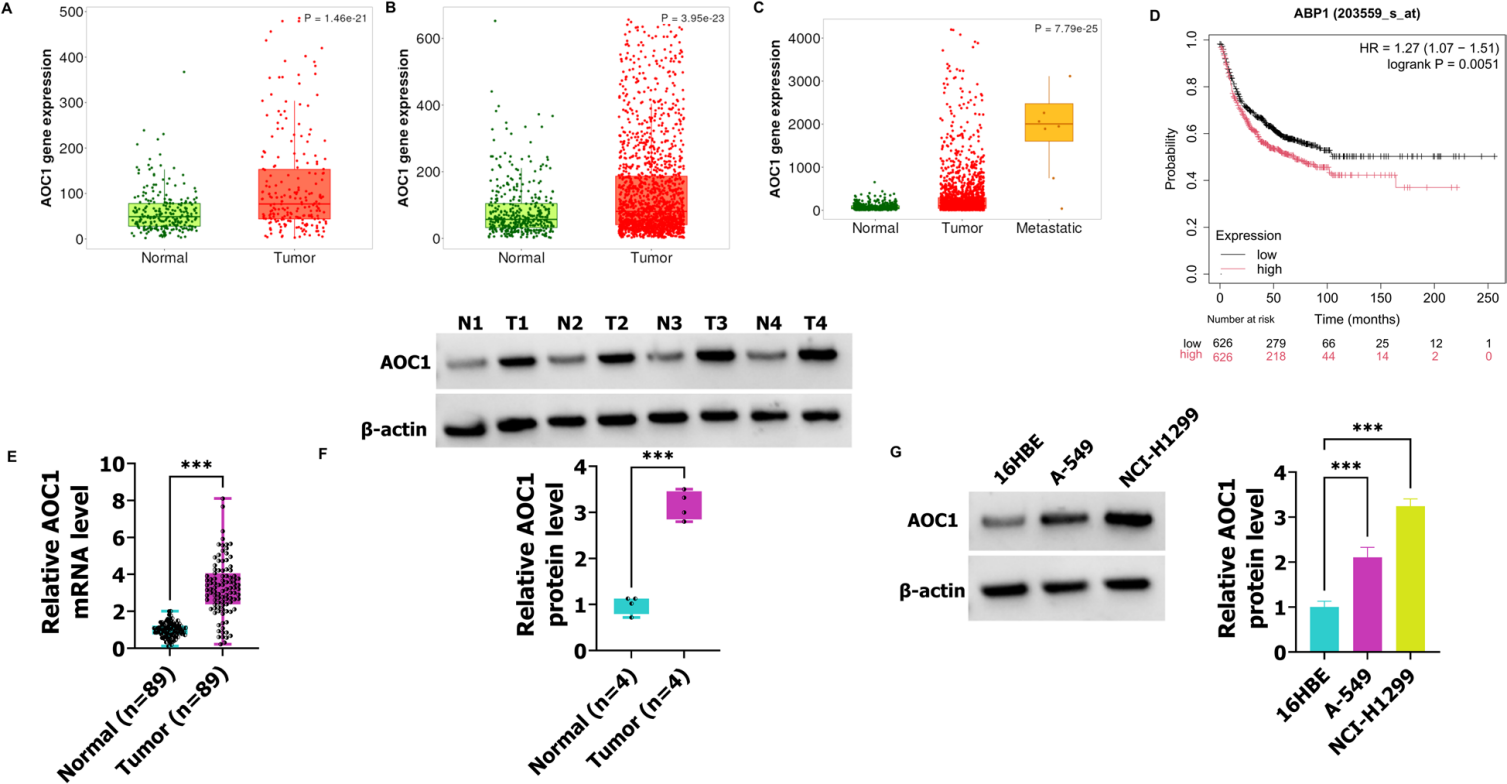
In NSCLC, AOC1 is highly expressed. **A**-**C** The TNMplot database was used to analyze AOC1 expression in NSCLC tissues and normal tissues. **D** The survival significance of high- or low-expression of AOC1 in NSCLC patients. **E** In NSCLC (*n* = 89) and normal tissues (*n* = 89), the AOC1 expression was detected using RT-qPCR. **F** The western blot was used to examine AOC1 levels in NSCLC (*n* = 4) and normal tissues (*n* = 4). **G** In 16HBE, A-549, and NCI-H1299 cells, the AOC1 levels were detected using western blot. *** *P* < 0.001

To identify the role of AOC1 in NSCLC, the AOC1 expression was analyzed in NSCLC tissues based on the TNMplot suites. As shown in Fig. 1A and B, the AOC1 levels were highly expressed in NSCLC tissues. Then, researchers found that AOC1 expression was noticeably higher in NSCLC metastasis tissues than in primary tumor tissues (Fig. 1C). As shown in Fig. 1D, elevated AOC1 levels were associated with poor progression in NSCLC patients. The RT-qPCR results demonstrated that AOC1 was increased in NSCLC tissues when compared with the Normal group (Normal (*n* = 89) and Tumor (*n* = 89)) (Fig. 1E). Similar results were obtained in western blot assay, namely, AOC1 expression was promoted in NSCLC tissues (Normal (*n* = 4) and Tumor (*n* = 4)) (Fig. 1F). Besides, the AOC1 was significantly higher in A-549 and NCI-H1299 cells than in 16HBE cells (Fig. 1G). Thus, these findings suggest that AOC1 expression is related to NSCLC.

### In A-549 and NCI-H1299 cells, AOC1 promotes proliferation, migration, and mitochondrial membrane potential and inhibits cell apoptosis and ROS levels

**Fig. 2:**
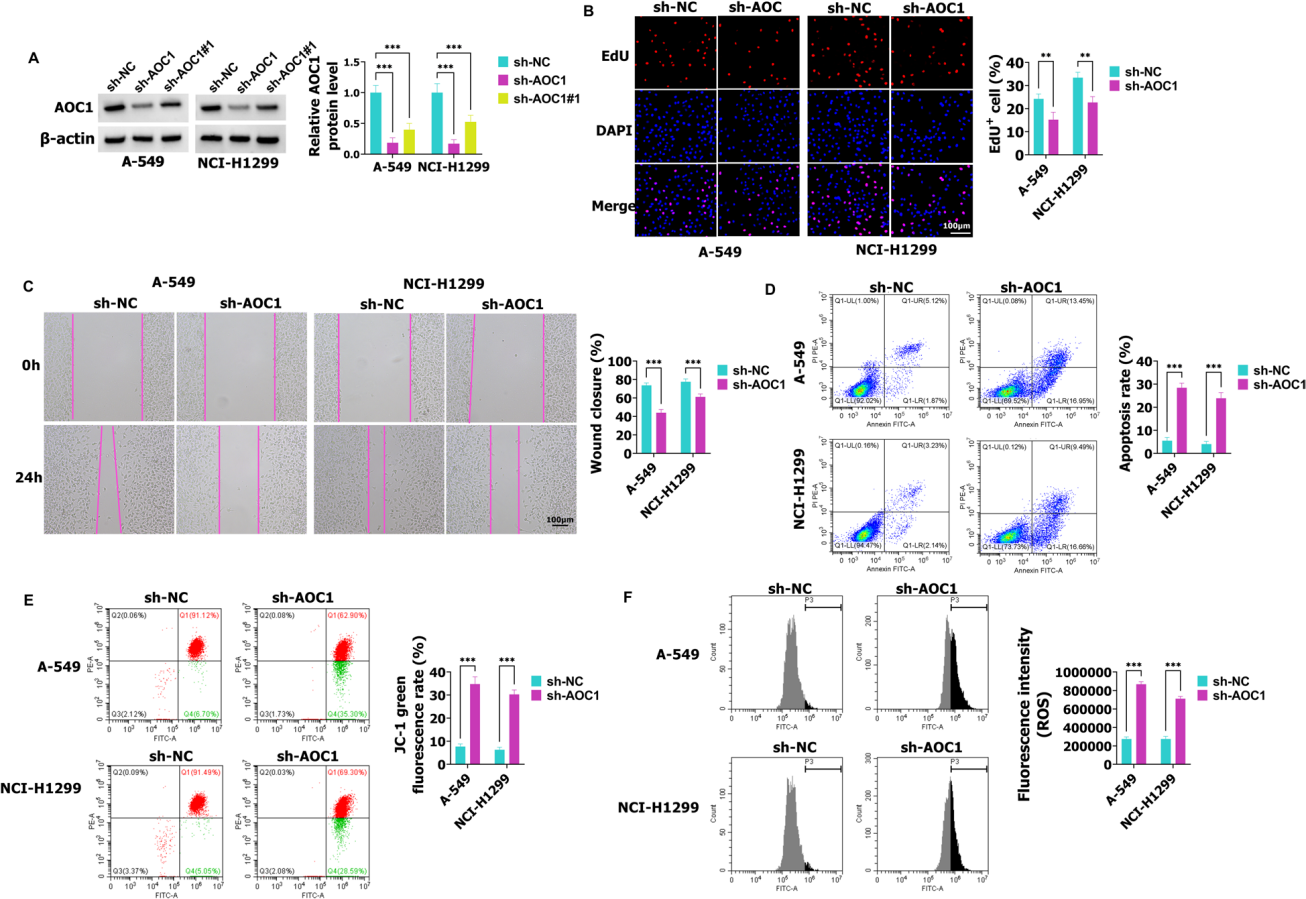
AOC1 facilitates A-549 and NCI-H1299 cell proliferation, and migration, and mitochondrial membrane potential, and hinders cell apoptosis and ROS levels. **A** The knockdown efficiencies of AOC1 were confirmed by western blot. **B**-**C** In A-549 and NCI-H1299 cells with sh-AOC1 transfection, the proliferation and migration abilities were analyzed using EdU and wound healing assays, respectively. **D** After A-549 and NCI-H1299 cells were transfected with sh-AOC1 or sh-NC, the cell apoptosis was detected using flow cytometry. **E**–**F** The mitochondrial membrane potential and ROS levels were examined using the JC-1 detection kit and flow cytometry, respectively. ** *P* < 0.01, *** *P* < 0.001

To evaluate the effect of AOC1 in NSCLC malignant behaviors, a specific shRNA against AOC1 was transfected into A-549 and NCI-H1299 cells, which was assessed by western blot. As shown in Fig. 2A, AOC1 levels were inhibited in sh-AOC1 and sh-AOC1#1 groups when compared with the sh-NC group, and the sh-AOC1 was selected for AOC1 knockdown experiments. The EdU assay (Fig. 2B) and wound healing assay (Fig. 2C), reflected the proliferation and migration ability, the results also showed that silencing AOC1 suppressed A-549 and NCI-H1299 cell proliferation and migration. However, overexpression of AOC1 promoted A-549 and NCI-H1299 cell proliferation and migration (Fig. S1A and B). In A-549 and NCI-H1299 cells, the cell apoptosis was promoted by sh-AOC1 (Fig. 2D). On the contrary, AOC1 overexpression inhibited A-549 and NCI-H1299 cell apoptosis (Fig. S1C). As shown in Fig. 2E, the mitochondrial membrane potential was decreased in A-549 and NCI-H1299 cells with sh-AOC1 transfection. In A-549 and NCI-H1299 cells, AOC1 increased mitochondrial membrane potential when compared with the vector group (Fig. S1D). Compared with the sh-NC group, sh-AOC1 facilitated ROS levels (Fig. 2F). As shown in Fig. S1E, AOC1 overexpression suppressed ROS production (Fig. S1E). To sum up, silencing AOC1 inhibits A-549 and NCI-H1299 cell proliferation, migration, and mitochondrial membrane potential, and promotes cell apoptosis and ROS levels, but overexpression of AOC1 has the opposite effect.

### AOC1 knockdown inhibits the M2 polarization of macrophages

**Fig. 3:**
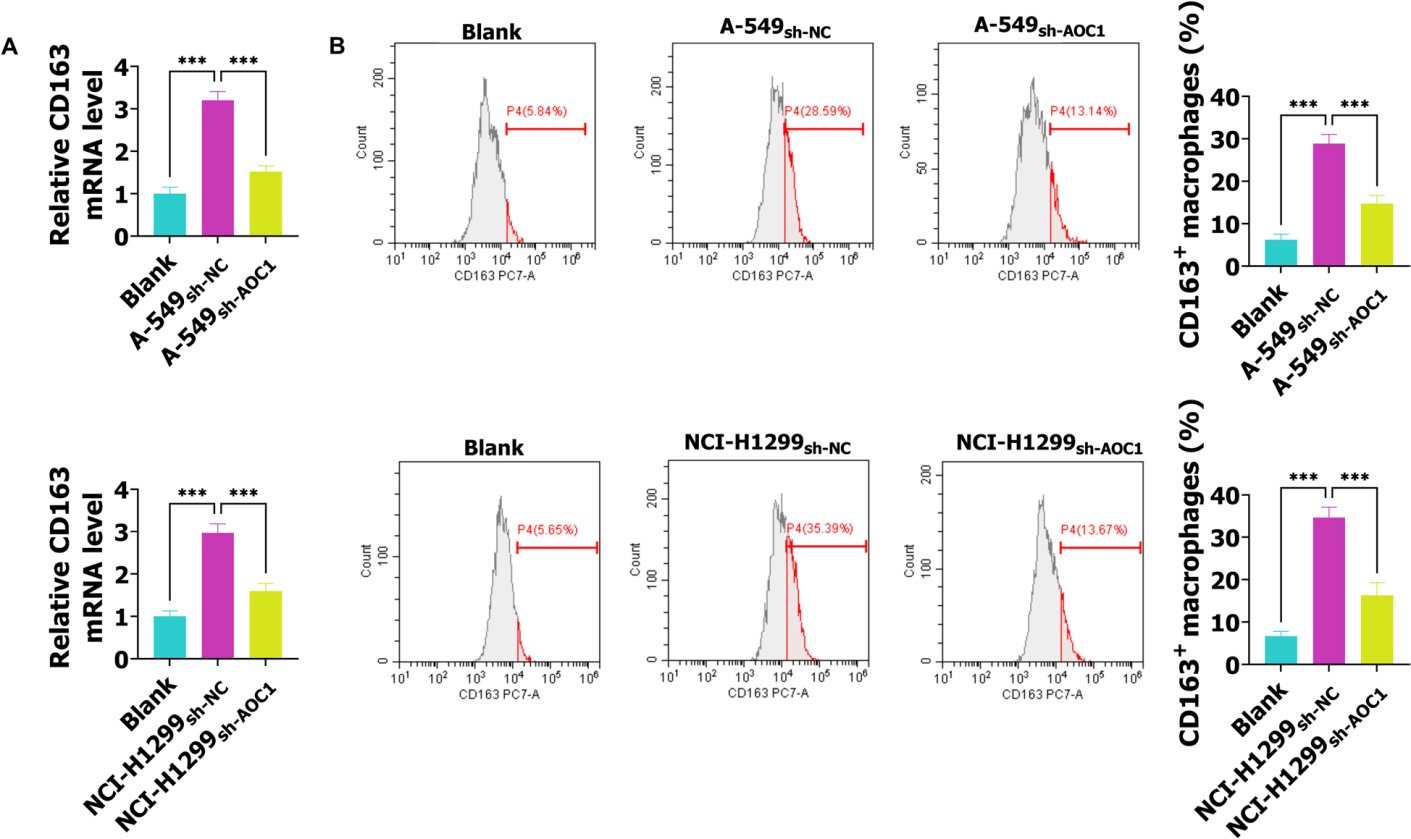
AOC1 knockdown inhibits the M2 polarization of macrophages. The THP-1 cells were treated with PMA to induce THP-1-M0 cells, which were cultured with the normal medium or medium collected from the A-549 or NCI-H1299 cells transfected with sh-NC, and sh-AOC1. **A** The CD163 levels were tested via RT-qPCR. **B** The flow cytometry was used to analyze the CD163.^+^ macrophages. *** *P* < 0.001

Macrophages are an important part of the immune system, and macrophage M2 polarization could facilitate malignant behaviors of tumors, including cell proliferation, invasion, and metastasis [16, 17]. Next, this study explored the relationship between AOC1 and macrophage M2 polarization. As shown in Fig. 3A, the RT-qPCR results demonstrated that CD163 levels were promoted in the A-549_sh-NC_ and NCI-H1299_sh-NC_ groups, while sh-AOC1 abolished this action. Besides, the flow cytometry results were consistent with the RT-qPCR, namely, M2 polarization marker CD163^+^ cell rate was increased in THP-1-M0 cells after co-culture with the medium of A-549 and NCI-H1299 cells transfected with sh-NC when compared with the Blank group, while silencing AOC1 abrogated this action (Fig. 3B). After A-549 and NCI-H1299 cells were transfected with AOC1, the CD163 mRNA expression was promoted when compared with the A-549_vector_ and NCI-H129_vector_ groups (Fig. S1F). Taken together, sh-AOC1 hinders M2 polarization of macrophages, and AOC1 overexpression promotes M2 polarization of macrophages.

### Silencing AOC1 hinders xenograft tumor growth in vivo

**Fig. 4:**
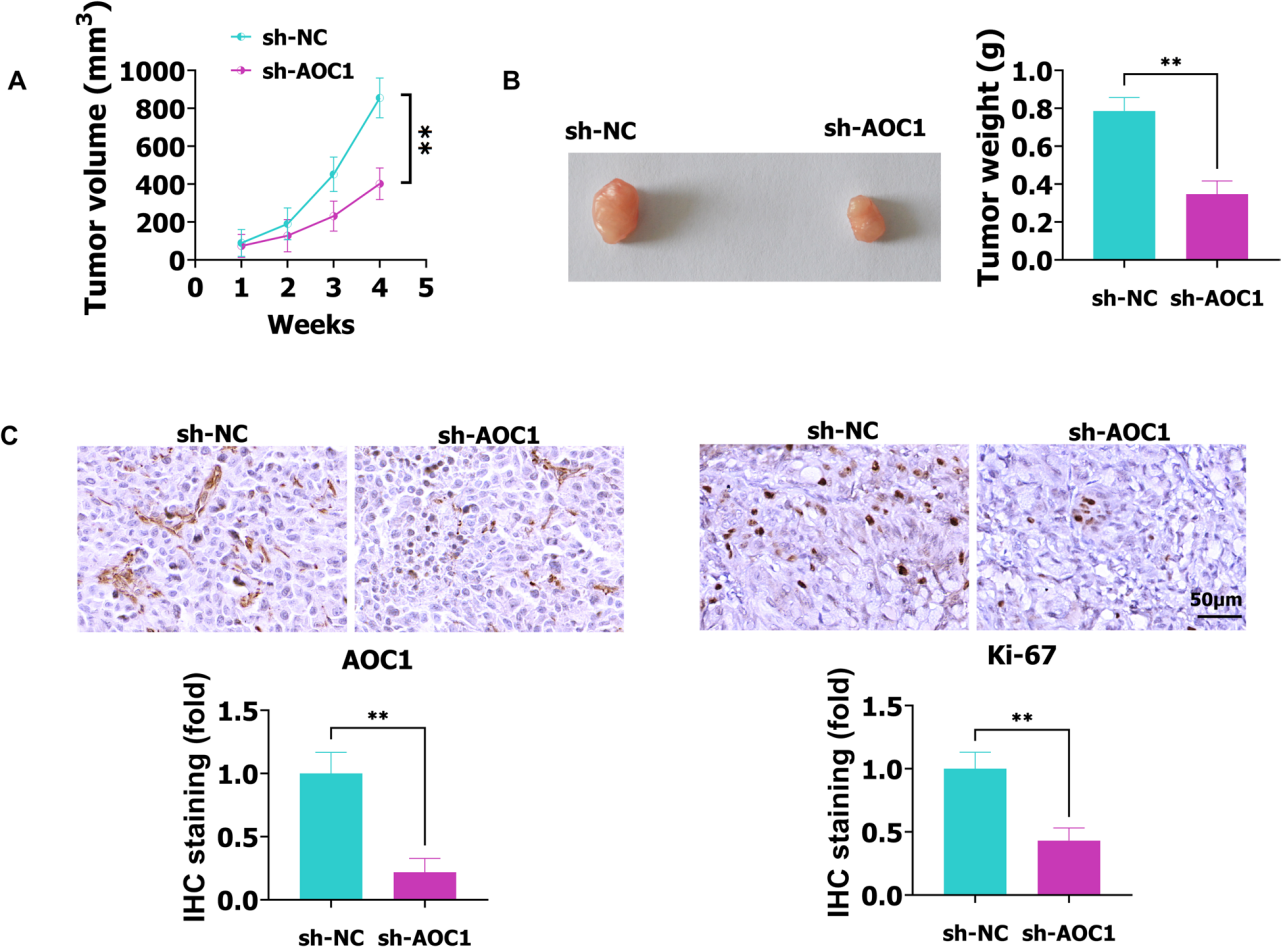
Knockdown of AOC1 inhibits xenograft tumor growth in vivo. **A**-**B** The tumor volume and weight in sh-NC and sh-AOC1 groups were analyzed. **C** In sh-NC and sh-AOC1 groups, the AOC1 and Ki-67 levels were detected using IHC assay. ** *P* < 0.01

To explore the biological function of AOC1 in tumor growth in vivo, a xenograft assay was performed by injecting paired tumor cells into nude mice. As shown in Fig. 4A, the tumor volume was inhibited by sh-AOC1 in a time-dependent manner. However, up-regulation of AOC1 promoted tumor volume and was positively related to time (Fig. S1G). Silencing AOC1 suppressed tumor weight of NSCLC (Fig. 4B), but AOC1 increased the tumor weight of NSCLC (Fig. S1H). Furthermore, the IHC assay showed that AOC1 and Ki-67 levels were decreased in the sh-AOC1 group compared with the sh-NC group (Fig. 4C). In summary, AOC1 promotes tumor growth.

### AOC1 transcription is activated by TFAP2A

**Fig. 5:**
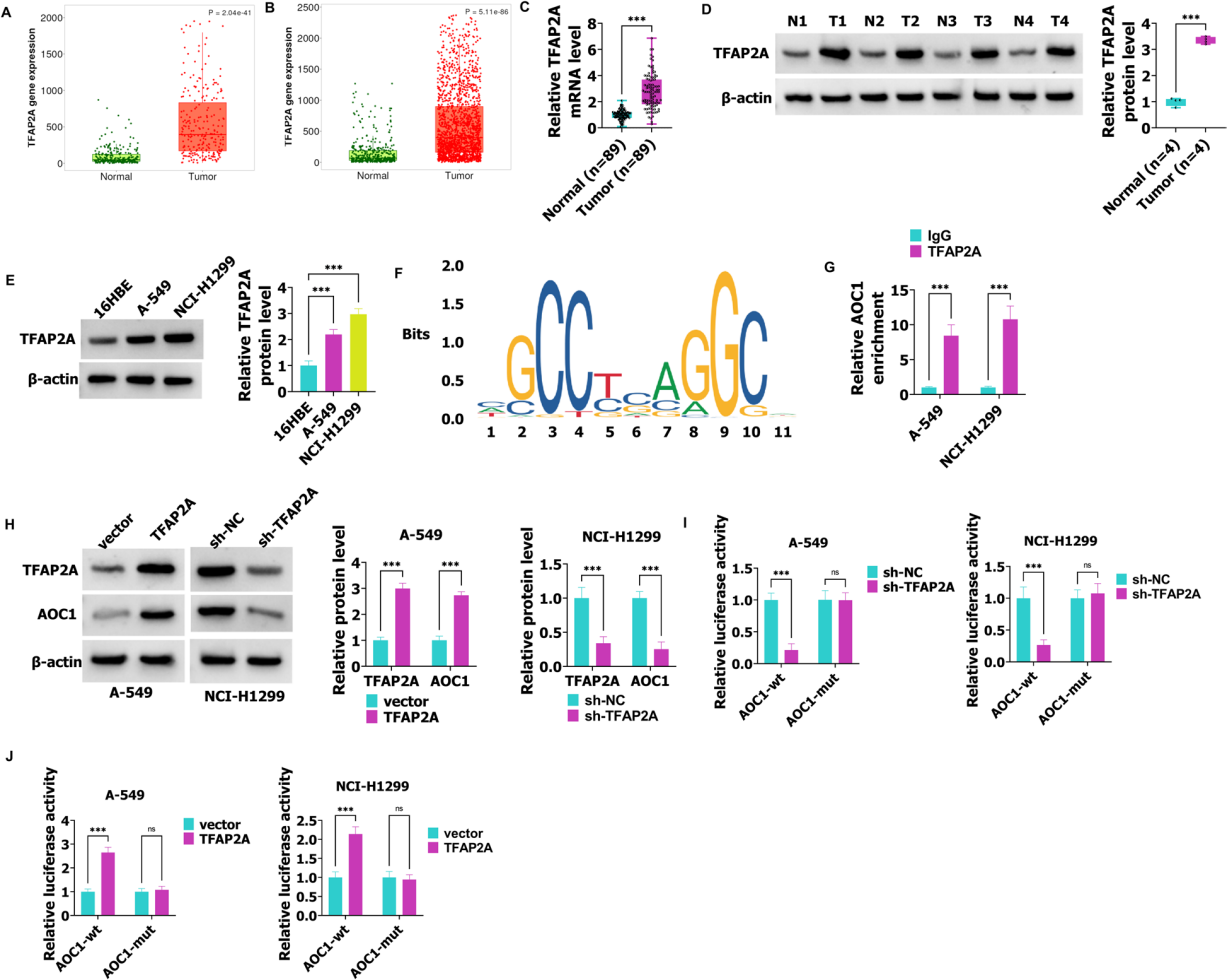
TFAP2A activates AOC1 transcription. **A**-**B** TFAP2A expression in NSCLC tissues and normal tissues was analyzed using the TNMplot database. **C** In NSCLC (*n* = 89) and normal tissues (*n* = 89), the TFAP2A levels were detected using RT-qPCR. **D** The western blot was used to examine TFAP2A expression in NSCLC (*n* = 4) and normal tissues (*n* = 4). **E** The TFAP2A expression in 16HBE, A-549, and NCI-H1299 cells was analyzed using western blot. **F** The JASPAR database was used to predict the binding sites between the AOC1 promoter and TFAP2A. **G** CHIP was used to determine the interaction between AOC1 and TFAP2A. **H** After A-549 and NCI-H1299 cells were transfected with vector/TFAPA2 and sh-NC/sh-TFAPA2, respectively, the AOC1 and TFAP2A levels were tested using western blot. **I**-**J** Luciferase activity was examined by dual luciferase reporter gene assay. *** *P* < 0.001 and ns indicated no difference

As shown in Fig. 5A and B, TFAP2A was increased in NSCLC tissues. Besides, the RT-qPCR and western blot results demonstrated that TFAP2A was highly expressed in tumor tissues compared with the normal tissues (Fig. 5C, D). In A-549 and NCI-H1299 cells, TFAP2A expression was promoted (Fig. 5E). Furthermore, the JASPAR database predicted that TFAP2A could bind with AOC1 promoter (Fig. 5F). Compared with the IgG group, AOC1 was significantly enriched in the TFAP2A group (Fig. 5G). Western blot showed that TFAP2A overexpression in A-549 cells promoted the expression of TFAP2A and AOC1, but silencing TFAP2A in NCI-H1299 cells hindered TFAP2A and AOC1 expression (Fig. 5H). After A-549 and NCI-H1299 cells were co-transfected with sh-TFAP2A and AOC1-wt, the luciferase activity was inhibited, but luciferase activity did not later when A-549 and NCI-H1299 cells were co-transfected with sh-TFAP2A and AOC1-mut (Fig. 5I). As shown in Fig. 5J, the luciferase activity was promoted in A-549 and NCI-H1299 cells with AOC1-wt and TFAP2A co-transfection, but the luciferase activity did not change after A-549 and NCI-H1299 cells were transfected with AOC1-mut and TFAP2A (Fig. 5J). To sum up, TFAP2A promotes AOC1 expression in NSCLC.

### TFAP2A facilitates the progression of NSCLC by regulating the expression of AOC1

**Fig. 6:**
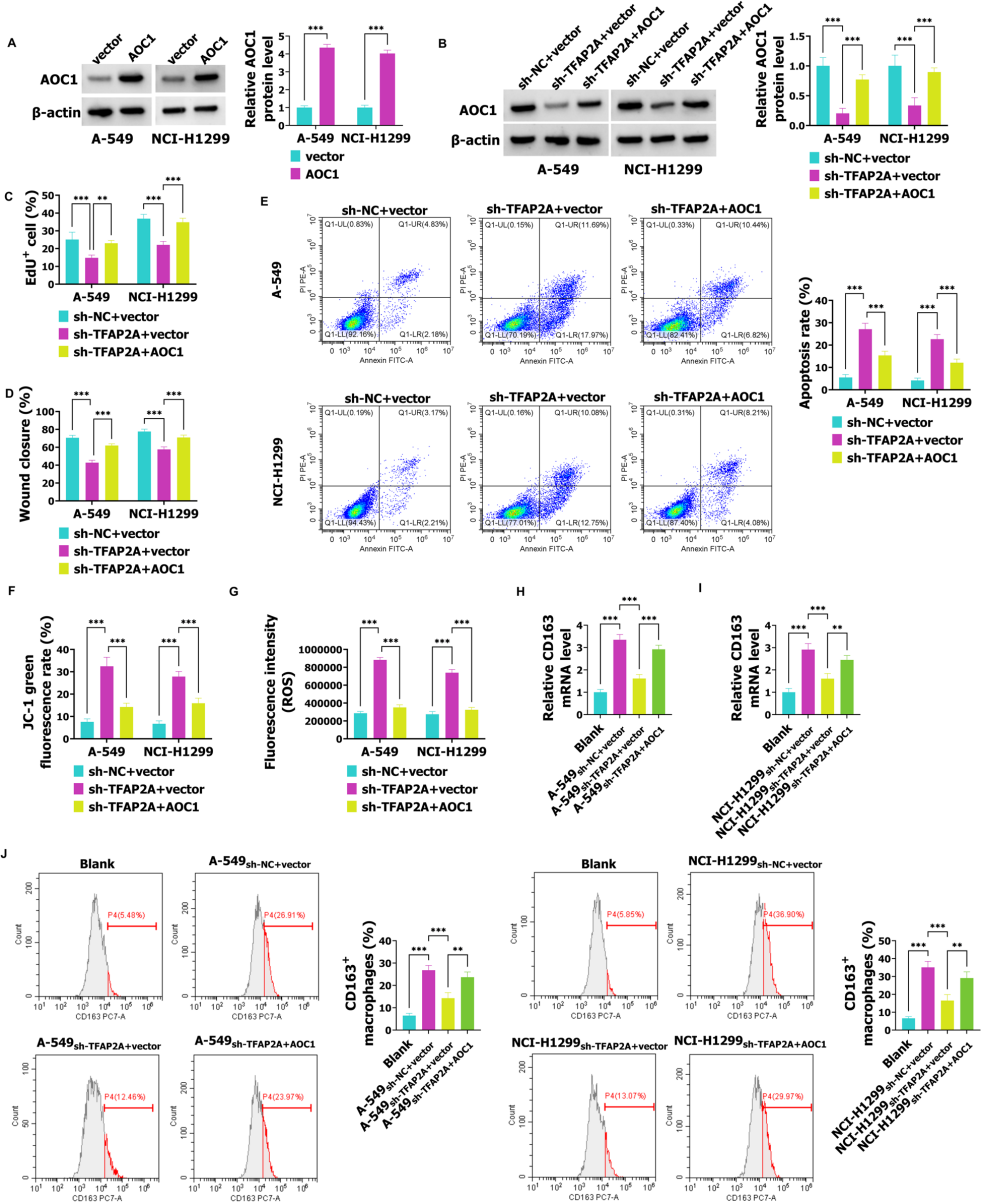
TFAP2A promotes NSCLC progression via regulating AOC1. **A** After A-549 and NCI-H1299 cells were transfected with vector or AOC1, western blot was used to analyze AOC1 expression. **B** The AOC1 levels were detected in sh-NC + vector, sh-TFAP2A + vector, and sh-TFAP2A + AOC1 groups by western blot. **C**-**E** In sh-NC + vector, sh-TFAP2A + vector, and sh-TFAP2A + AOC1 groups, the proliferation, migration, and apoptosis ability was detected using EdU, wound healing, and flow cytometry assays, respectively. **F**-**G** The mitochondrial membrane potential and ROS were examined using JC-1 and ROS detection kits. **H**-**J**: The THP-1 cells were treated with PMA to induce THP-1-M0 cells, which were cultured with the normal medium or medium collected from the A-549 or NCI-H1299 cells co-transfected with sh-NC + vector, sh-TFAP2A + vector, or sh-TFAP2A + vector + AOC1. **H**-**I** The CD163 levels in each group were analyzed using RT-qPCR. **J** The flow cytometry was employed to examine CD163.^+^ macrophages in each group. ** *P* < 0.01, *** *P* < 0.001

Above results demonstrated that AOC1 promoted the malignant behaviors of NSCLC, and TFAP2A could promote AOC1 transcription. Besides, TFAP2A promoted NSCLC progression [18]. Thus, researchers suggested that TFAP2A could regulate AOC1 to facilitate malignant behaviors of NSCLC. As shown in Fig. 6A, overexpression of AOC1 promoted AOC1 expression in A-549 and NCI-H1299 cells. AOC1 up-regulation abolished sh-TFAP2A-inhibited expression of AOC1 (Fig. 6B). Compared with the sh-NC + vector group, sh-TFAP2A suppressed cell proliferation and migration, while this action was weakened by AOC1 (Fig. 6C, D). In the TFAP2A + sh-NC group, the cell proliferation and migration was promoted when compared with the vector + sh-NC, which was weakened by sh-AOC1 (Fig. S2A and B). Overexpression of AOC1 weakened sh-TFAP2A-regulated facilitation of apoptosis (Fig. 6E). When compared with the vector + sh-NC group, the cell apoptosis was reduced in the TFAP2A + sh-NC group, while silencing AOC1 abolished this action (Fig. S2C). Compared with the sh-NC + vector group, down-regulated TFAP2A inhibited mitochondrial membrane potential and elevated ROS, which was abolished by AOC1 (Fig. 6F, G). The JC-1 green fluorescence rate and ROS was inhibited in the TFAP2A + sh-NC group compared with the vector + sh-NC group, but this action was reversed by sh-AOC1 (Fig. S2D and E). Compared with the Blank group, CD163 expression was facilitated in the A-549_sh-NC+vector_ and NCI-H1299_sh-NC+vector_ groups, while this effect was weakened by sh-TFAP2A; besides, AOC1 abrogated sh-TFAP2A-inhibited CD163 levels (Fig. 6H, I). Similar results were obtained in flow cytometry assay, namely, M2 polarization of macrophages was promoted in THP-1-M0 cells after co-culture with the medium of A-549 and NCI-H1299 cells transfected with sh-NC and vector when compared with the Blank group, while sh-TFAP2A abrogated this action; AOC1 overexpression abolished sh-TFAP2A-regulated suppression of CD163^+^ macrophages (Fig. 6J). However, the CD163 mRNA expression was promoted in A-549_vector+sh-NC_ and NCI-H1299_vector+sh-NC_ groups, which was enhanced by TFAP2A; when compared with the A-549_TFAP2A+sh-NC_ and NCI-H1299_TFAP2A+sh-NC_ groups, the expression of CD163 was reduced in the A-549_TFAP2A+sh-AOC1_ and NCI-H1299_TFAP2A+sh-AOC1_ groups (Fig. S2F and G). Overall, silencing TFAP2A hinders malignant behaviors of NSCLC by regulating AOC1.

## Discussion

Studies have shown that NSCLC is one of the most common types of lung cancer and is the cause of most cancer-related deaths worldwide [19]. Treatment for patients with NSCLC includes surgery, radiation, chemotherapy, targeted therapies, and immunotherapy [20, 21]. Despite significant advances in targeted therapies and immunotherapy, the 5-year survival rate for all stages of NSCLC remains about 28% [22, 23]. In addition, late diagnosis of NSCLC often leads to a poor prognosis [24]. Therefore, there is an urgent need to find new potential therapeutic targets for NSCLC. In almost all types of cancer, polyamines (putrescine, spermidine and spermine) levels were increased, and they play a crucial role in the proliferation of tumors [25]. Spermidine/spermine N1-acetyltransferase (SSAT-1) is a key regulator of polyamine homeostasis; its regulation reduces intracellular polyamine levels, leading to cell cycle arrest [26]. However, mammalian cells can restore polyamine pools through the action of polyamine oxidase (PAOX), which converts acetyl-spermine back to spermidine and acetyl-spermidine to putrescine [27]. Notably, indomethacin induced SSAT-1 expression via the nucleolin-CDK1 axis and exhibited synergistic anticancer effects with the PAOX inhibitor methoctramine in lung cancer cells [28]. Additionally, AOC1, a crucial enzyme in polyamine metabolism, has been implicated as an oncogene in various types of tumors[29]. However, the specific role of amine oxidase like AOC1 in lung cancer is still less studied.

Hu et al*.* showed that methyltransferase-like 14 (METTL14) could boost the development of nasopharyngeal carcinoma (NPC) cells partly via regulating AOC1 mRNA stability [30]. In addition, the expression of AOC1 was reported to be higher in GC tissues than in tissues of normal and noncancerous, and AOC1 promoted GC progression in humans [8]. We suggest that AOC1 may be an oncogenic factor in NSCLC. In our study, the results indicated that AOC1 was elevated in NSCLC tissues and cells, which suggested that AOC1 was related to poor progression of NSCLC. In colorectal cancer (CRC) tissues, AOC1 levels were promoted, and the functional experiments demonstrated that AOC1 silence retarded the migration and proliferation of CRC cells [31]. Our findings showed that AOC1 promoted NSCLC malignant progression in vitro and in vivo.

Macrophages, due to their high plasticity and heterogeneity, are an important component of the tumor microenvironment (TME) [32]. Additionally, macrophages are an important part of the immune system, and macrophage M2 polarization can facilitate malignant behaviors of tumors, including cell proliferation, invasion, and metastasis [16, 17]. The positive expression of AOC1 was significantly associated with poor clinical outcomes of colorectal cancer and promoted the tumor cell invasion phenotype by inducing the epithelial-mesenchymal transition (EMT) pathway [31]. Xu et al*.* demonstrated that AOC1 promoted tumor progression by facilitating the EMT pathway in gastric cancer [8]. In this study, knockdown of AOC1 repressed macrophage M2 polarization and the results suggested that AOC1 may be a tumorigenic factor in NSCLC and regulate the TME remodeling via macrophage polarization. Additionally, the JASPAR database showed that TFAP2A had a binding site on AOC1 promoter. Based on these findings, researchers suggest that oncogenic action of AOC1 in NSCLC may be regulated by TFAP2A.

TFAP2 interacted with the tumor microenvironment by regulating angiogenesis and reshaping the immune microenvironment [33]. TFAP2A is a transcription factor that coordinates a variety of cellular processes. TFAP2A could facilitate NPC development in vivo and in vitro [34]. TFAP2A derived melanoma metastasis by regulating E2F and EZH2 [35]. TFAP2A was related to worse clinical stage and prognosis in bladder cancer patients [36]. In addition, TFAP2A was increased in NSCLC, and overexpression of TFAP2A enhanced NSCLC cell invasion, migration, and proliferation [18]. This study first demonstrated that TFAP2A knockdown inhibited NSCLC progression via regulating AOC1 transcription. Heterogeneous nuclear ribonucleoprotein C could promote collagen fiber alignment and immune escape in breast cancer by activating vir like m6A methyltransferase associated-mediated TFAP2A/discoidin domain receptor tyrosine kinase 1 axis[37]. TFAP2A was involved in regulating the function of tumor-associated macrophages in CRC [38]. Researchers speculated that TFAP2A was involved in macrophage polarization in NSCLC. In our study, AOC1 abolished sh-TFAP2A-regulated inhibition of macrophage M2 polarization, which was consistent with the previous finding that TFAP2A could enhance M2 polarization of macrophages in liver cancer [39]. Therefore, in NSCLC, TFAP2A may be an upstream target for AOC1.

To sum up, this study first reveals that TFAP2A can contribute to NSCLC progression via promoting AOC1 expression. However, the study has some limitations. For example, this study overly relied on a single cell line, and lacked dynamic live-cell imaging techniques to capture real-time movement changes. But our findings provide novel insights into the molecular mechanisms for NSCLC progression, and AOC1 and TFAP2A may be potential targets for NSCLC treatment.

## Supplementary Information

Supplementary Material 1. Fig. S1 AOC1 promotes NSCLC tumor growth in vivo and in vitro. A-E: The A-549 and NCI-H1299 cells were transfected with vector and AOC1. (A) The cell proliferation was analyzed using EdU assays (B-C) The cell migration and apoptosis abilities were examined using wound healing and flow cytometry. (D-E) The mitochondrial membrane potential and ROS levels were detected using the JC-1 detection kit and flow cytometry, respectively. (F) The THP-1 cells were treated with PMA to induce THP-1-M0 cells, which were cultured with the normal medium or medium collected from the A-549 or NCI-H1299 cells transfected with vector, and AOC1. The CD163 levels were examined using RT-qPCR. (G-H) The tumor volume and weight in vector and AOC1 groups were analyzed. * *P* < 0.05, ** *P* < 0.01, and *** *P* < 0.001.

Supplementary Material 2. Fig. S2 TFAP2A promotes NSCLC progression via regulating AOC1. A-E: The A-549 and NCI-H1299 cells were transfected with vector + sh-NC, TFAP2A + sh-NC, or TFAP2A + sh-AOC1. (A-B) The EdU and wound healing were used to analyze the cell proliferation and migration. (C) The cell apoptosis was analyzed using flow cytometry. (D) The JC-1 green fluorescence rate was examined using JC-1 detection kit. (E) Flow cytometry was used to examine the ROS levels. (F-G) In the Blank, A-549_vector+sh-NC_, NCI-H1299_vector+sh-NC_, A-549_TFAP2A+sh-NC_, NCI-H1299_TFAP2A+sh-NC_, A-549_TFAP2A+sh-AOC1_, and NCI-H1299_TFAP2A+sh-AOC1_ groups, the CD163 mRNA expression was detected using RT-qPCR. * *P* < 0.05, ** *P* < 0.01, and *** *P* < 0.001.

## Data Availability

The data are available from the corresponding author upon reasonable request.
