# Supplementary figures and images for: TFAP2A promotes NSCLC malignant progression by enhancing AOC1 transcription
Source: Hereditas. 2025 Aug 14;162:156. doi: 10.1186/s41065-025-00524-2 (PMC12351806; doi:10.1186/s41065-025-00524-2)

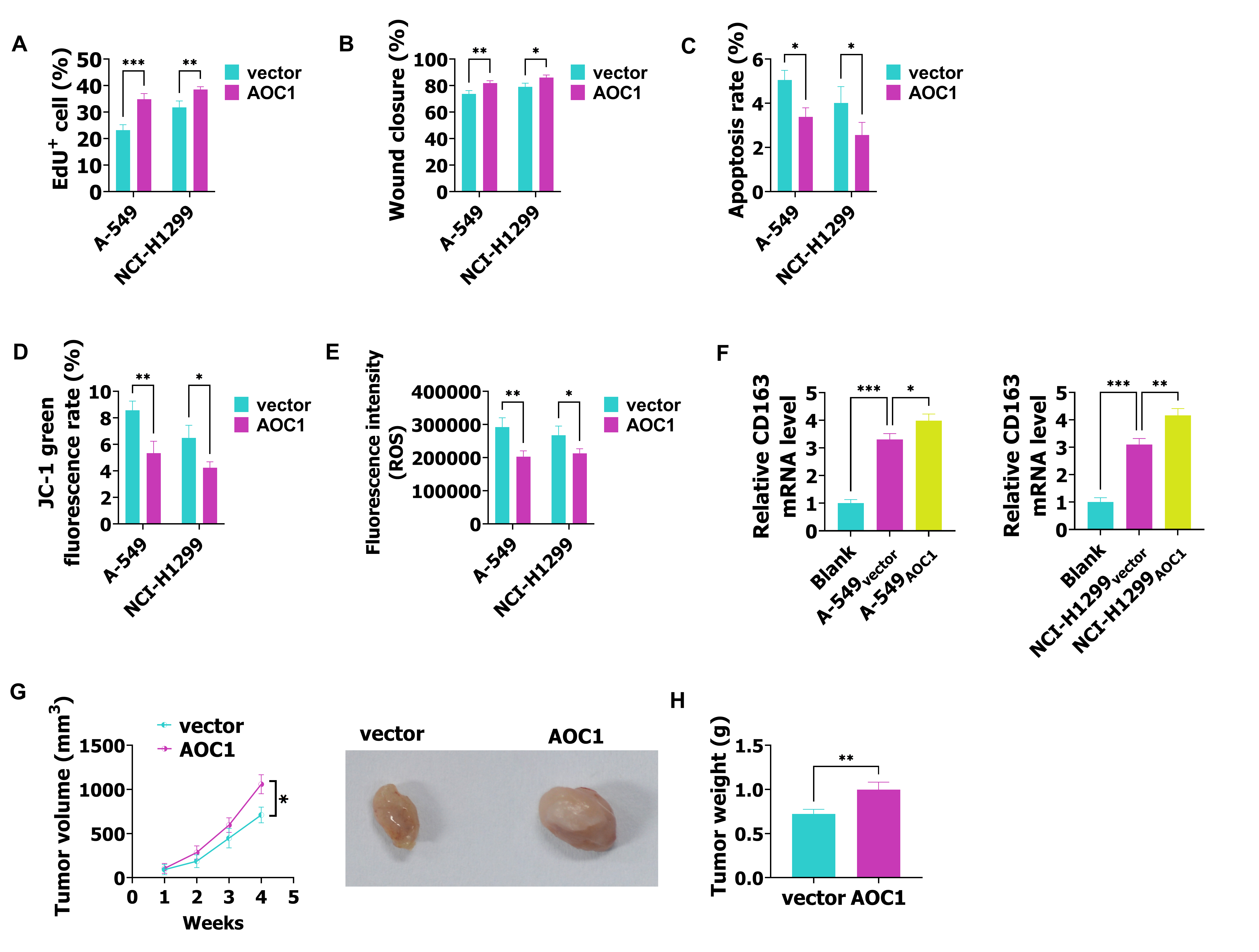

Supplement: Supplementary file 1 — Supplementary Material 1. Fig. S1 AOC1 promotes NSCLC tumor growth in vivo and in vitro. A-E: The A-549 and NCI-H1299 cells were transfected with vector and AOC1. (A) The cell proliferation was analyzed using EdU assays (B-C) The cell migration and apoptosis abilities were examined using wound healing and flow cytometry. (D-E) The mitochondrial membrane potential and ROS levels were detected using the JC-1 detection kit and flow cytometry, respectively. (F) The THP-1 cells were treated with PMA to induce THP-1-M0 cells, which were cultured with the normal medium or medium collected from the A-549 or NCI-H1299 cells transfected with vector, and AOC1. The CD163 levels were examined using RT-qPCR. (G-H) The tumor volume and weight in vector and AOC1 groups were analyzed. * P < 0.05, ** P < 0.01, and *** P < 0.001. [file 41065_2025_524_MOESM1_ESM.tif]

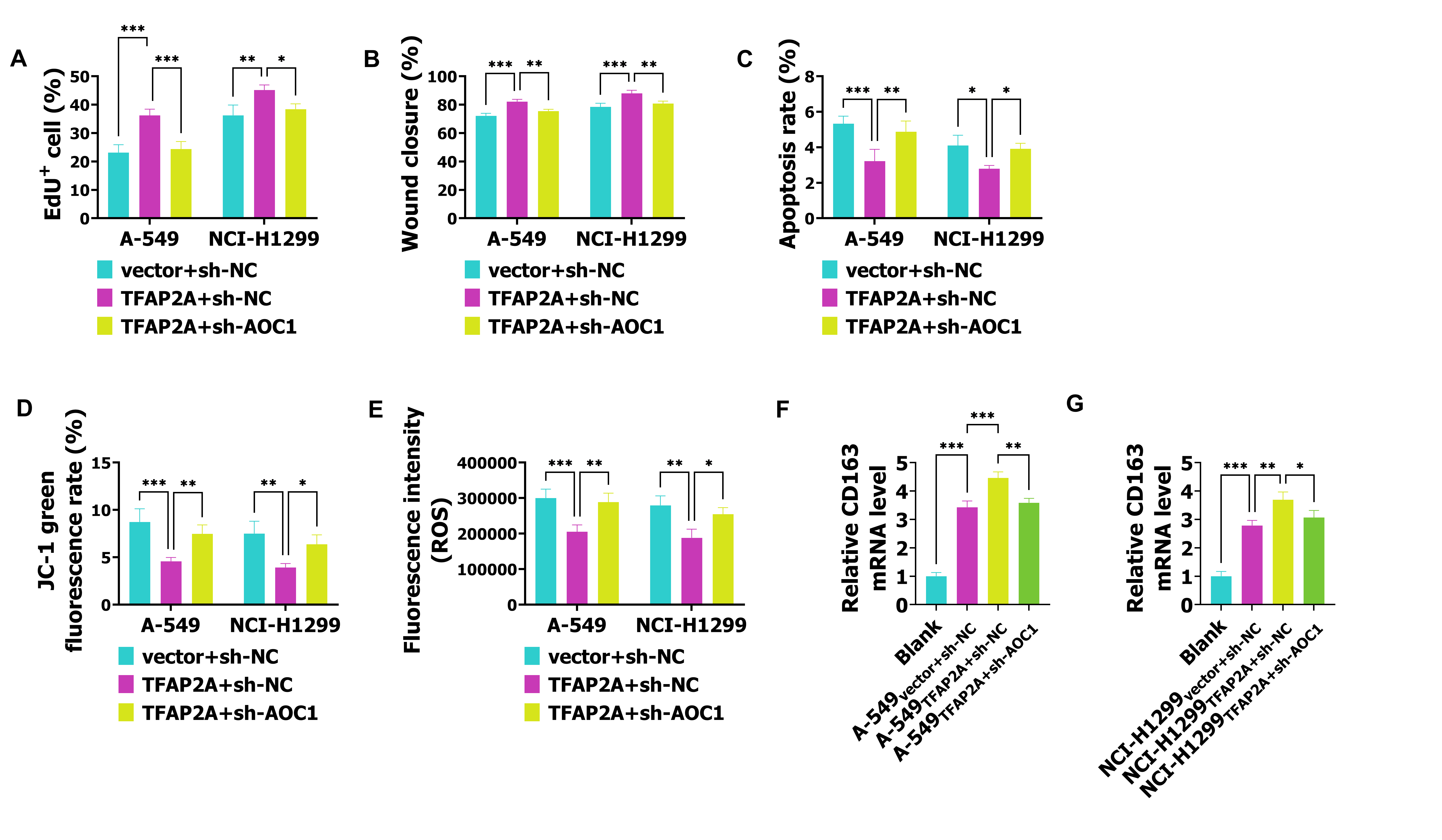

Supplement: Supplementary file 2 — Supplementary Material 2. Fig. S2 TFAP2A promotes NSCLC progression via regulating AOC1. A-E: The A-549 and NCI-H1299 cells were transfected with vector + sh-NC, TFAP2A + sh-NC, or TFAP2A + sh-AOC1. (A-B) The EdU and wound healing were used to analyze the cell proliferation and migration. (C) The cell apoptosis was analyzed using flow cytometry. (D) The JC-1 green fluorescence rate was examined using JC-1 detection kit. (E) Flow cytometry was used to examine the ROS levels. (F-G) In the Blank, A-549vector+sh-NC, NCI-H1299vector+sh-NC, A-549TFAP2A+sh-NC, NCI-H1299TFAP2A+sh-NC, A-549TFAP2A+sh-AOC1, and NCI-H1299TFAP2A+sh-AOC1 groups, the CD163 mRNA expression was detected using RT-qPCR. * P < 0.05, ** P < 0.01, and *** P < 0.001. [file 41065_2025_524_MOESM2_ESM.tif]
